# The Bias and Signal Attenuation Present in Conventional Pollen-Based Climate Reconstructions as Assessed by Early Climate Data from Minnesota, USA

**DOI:** 10.1371/journal.pone.0113806

**Published:** 2015-01-20

**Authors:** Jeannine-Marie St. Jacques, Brian F. Cumming, David J. Sauchyn, John P. Smol

**Affiliations:** 1 Prairie Adaptation Research Collaborative, University of Regina, Regina, Saskatchewan, Canada, S4S 7H9; 2 Paleoecological Environmental Assessment and Research Lab, Department of Biology, Queen’s University, Kingston, Ontario, Canada, K7L 3N6; Ecole Pratique des Hautes Etudes, FRANCE

## Abstract

The inference of past temperatures from a sedimentary pollen record depends upon the stationarity of the pollen-climate relationship. However, humans have altered vegetation independent of changes to climate, and consequently modern pollen deposition is a product of landscape disturbance and climate, which is different from the dominance of climate-derived processes in the past. This problem could cause serious signal distortion in pollen-based reconstructions. In the north-central United States, direct human impacts have strongly altered the modern vegetation and hence the pollen rain since Euro-American settlement in the mid-19^th^ century. Using instrumental temperature data from the early 1800s from Fort Snelling (Minnesota), we assessed the signal distortion and bias introduced by using the conventional method of inferring temperature from pollen assemblages in comparison to a calibration set from pre-settlement pollen assemblages and the earliest instrumental climate data. The early post-settlement calibration set provides more accurate reconstructions of the 19^th^ century instrumental record, with less bias, than the modern set does. When both modern and pre-industrial calibration sets are used to reconstruct past temperatures since AD 1116 from pollen counts from a varve-dated record from Lake Mina, Minnesota, the conventional inference method produces significant low-frequency (centennial-scale) signal attenuation and positive bias of 0.8-1.7°C, resulting in an overestimation of Little Ice Age temperature and likely an underestimation of the extent and rate of anthropogenic warming in this region. However, high-frequency (annual-scale) signal attenuation exists with both methods. Hence, we conclude that any past pollen spectra from before Euro-American settlement in this region should be interpreted using a pre-Euro-American settlement pollen set, paired to the earliest instrumental climate records. It remains to be explored how widespread this problem is when conventional pollen-based inference methods are used, and consequently how seriously regional manifestations of global warming have been underestimated with traditional pollen-based techniques.

## Introduction

Both climate and landscape disturbance have important impacts on vegetation. Analysis of pollen in well-dated sediment cores has been used to estimate past changes in climate, but these relationships have been based on modern pollen-climate transfer functions. Due to large-scale landscape disturbance, the relationship between modern climate and pollen deposition is quite possibly different from the relationship between climate and pollen rain in the pre-industrial past. This largely overlooked problem is of concern because the inference of past climates from the sedimentary pollen record relies upon the pollen-climate relationship being constant in time from the calibration period to the reconstruction period ([1–3], but see [4–6]). Any assessment of the accuracy and bias of pollen-inferred climate records is important because of its potential applications, among which are more accurate high-resolution pollen-based climate reconstructions for the past millennium, and the validation of the accuracy of other proxy-based climate reconstructions, such as those from tree-rings over the past 2000 years (e.g., [7–11]).

There is considerable interest in temperature reconstructions of the last millennium and in the drivers of this natural climate variability, which are needed to assess the extent and rate of anthropogenic global warming and specifically the degree to which it exceeds natural climate variability (e.g., [8, 9, 11]). Paleoclimate reconstructions are also needed to evaluate whether climate models successfully simulate climate on multi-centennial timescales, and to strengthen our understanding of important feedbacks and mechanisms [2, 12, 13]. The many published reconstructions of hemispheric and global paleo-temperatures, using a wide variety of statistical techniques [2, 11], include high-resolution pollen paleoclimate assessments as important inputs [7, 9, 14]. Although these myriad northern hemispheric reconstructions generally agree on the timing of the Little Ice Age (LIA) and the Medieval Climate Anomaly (MCA), there is considerable variance in the estimation of the temperature differences between the extremes of the LIA and the MCA, between the extremes of the LIA and the late 20^th^ century warming, and between the extremes of the MCA and 20^th^ century warming, although there is accumulating confidence that late 20^th^ century warming exceeds that of the MCA [11]. However, these hemispheric millennial-length temperature records are only as reliable as the individual reconstructions on which that they are founded. Low-frequency variance can be attenuated in paleoclimate temperature reconstructions [15–17]. There have been calls for the scientists who generate the base reconstructions, whether based upon pollen, tree-rings, isotopes, boreholes, marine sediments, etc., to improve proxy validation and to critically examine how temperatures are inferred from their proxy data, and whether these relationships are indeed stationary and linear through the reconstruction periods as assumed [1, 12, 18–20].

In the American Midwest, logging, fire suppression, deforestation and agriculture have greatly changed vegetation composition and the composition of its pollen rain since Euro-American settlement in the mid-1800s. Minnesota is uniquely rich in early climate stations from military forts which predate large-scale Euro-American settlement [21], allowing a rare opportunity to test whether or not the pollen-climate relationship is stationary in time, as has generally been assumed. In this paper we assess the significance of low-frequency signal distortion and bias introduced by using a conventional modern pollen-climate calibration set versus an early post-settlement calibration set to infer temperature from a previously-published high-resolution pollen record from Lake Mina, Alexandria, Minnesota [5, 22]. Earlier work has established the utility of settlement era calibration datasets for this region [4, 5]. The reconstructions are compared to early climate records, beginning in AD 1820, from Fort Snelling (Minneapolis-St. Paul), Minnesota. The conventional inference method produces significant low-frequency (centennial-scale) signal attenuation and bias, resulting in an overestimation of LIA temperature and therefore an underestimation of the extent and rate of anthropogenic warming in this region. The early post-settlement calibration set provides more accurate reconstructions of the 19^th^ century instrumental record.

## Study Site, Datasets and Methods

Minnesota is a sensitive locale for paleoclimate reconstructions because it is quartered by two orthogonal continental-scale ecotones controlled by climate. The prairie-forest ecotone, with its wide transitional expanse of parkland, runs north-south, following the gradient in effective moisture (annual precipitation minus evapotranspiration) (modern Minnesota climate maps are shown in Fig. 1 of [5]). The forest itself is divided between the northern conifer-dominated boreal forest and the southern *Quercus*-dominated deciduous forest which closely follows the southern edge of USDA Climate Zone 3 (i.e., with an average winter minimum temperature of -34° to -40°C). The locations of these ecotones can be traced in pre-settlement vegetation patterns reconstructed from General Land Office surveys in Minnesota (AD 1847–1907) [[23]; Great Lakes Ecological Assessment http://www.ncrs.fs.fed.us/gla/histveg/images/mnorveg.gif, accessed June 28, 2013]. Hence, this sensitive location can be used to track paleoclimate signals from climate-driven shifts in these ecotones over at least the past several millennia.

**Figure 1:**
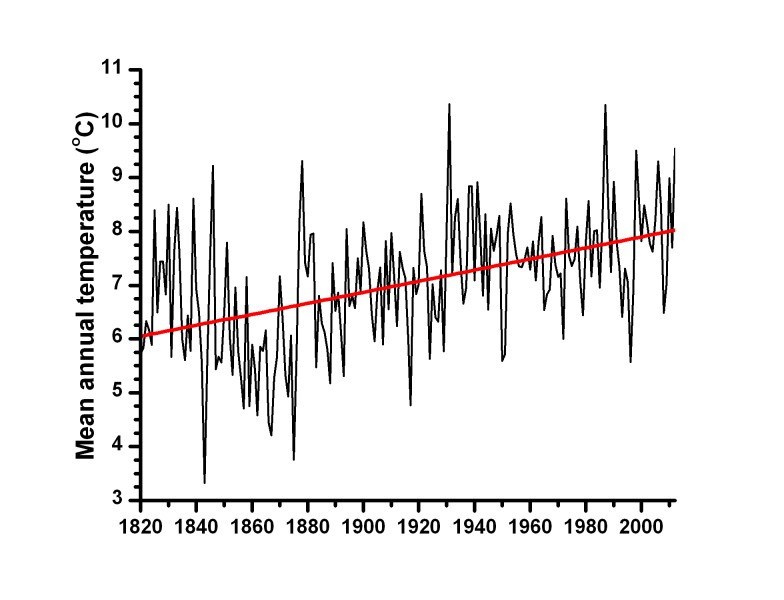
Fort Snelling (Minneapolis-St. Paul), Minnesota, historical record of mean annual temperature (°C) for AD 1820–2012. Red ordinary-least-squares regression line shows significant increase (*t* = 7.45, *d.f.* = 191, *p-value* = 3.5 × 10^–6^).

The early post-settlement pollen-climate calibration set is an extension of Cushing’s 100 BP pollen dataset [4], which consists of pollen samples immediately before the *Ambrosia* rise, which demarcates Euro-American settlement in Minnesota lake sediment cores. Landscape disturbance from settlement in Minnesota began only in the 1870s with the building of the railroads after the U.S. Civil War (AD 1861–1865) and the Sioux War of AD 1862. Earlier Native American Oneota Sioux maize-based agriculture tended to be localized and had relatively low landscape impacts in this area; although Native American agriculture and silviculture had pronounced landscape impacts in other regions [24, 25]. We extended the 100 BP pollen dataset using sites from the North American Pollen Database [http://www.ncdc.noaa.gov/paleo/pollen.html, accessed June 10, 2006], giving a total of 133 sites. We added AD 1895–1924 climate normals (the earliest geographically dense normals) from nearby early instrumental records [Minnesota State Climatology Office, http://climate.umn.edu/hidrius/rius.asp, accessed June 10, 2006] to produce a “pre-settlement” or “1870” calibration set [5]. We compiled a corresponding “modern” pollen-climate dataset compiled in the conventional fashion from core-top pollen samples and modern climate normals from AD 1961–1990. The distribution and qualitative changes of modern and pre-industrial pollen types was examined using response surfaces to assess the degree of vegetation change associated over the last c. 100 years due to climate and land-use changes. St. Jacques et al. [5] determined that February and May mean temperatures and effective moisture explained the greatest amount of pollen relative abundance variance, and were therefore the climate variables most suitable for climate reconstructions using transfer functions based upon two-component weighted averaging-partial least squares regression (WA-PLS2). We report here on the results for the temperature reconstructions. The effective moisture results will appear in a subsequent publication.

To assess the signal attenuation and bias of the modern and the pre-settlement pollen temperature transfer functions, we compared millennial-length temperature reconstructions from a high-resolution pollen record derived from Lake Mina in west-central Minnesota [5, 22, 26, 27] using both the pre-settlement and modern pollen-climate calibration sets, to the earliest instrumental climate records [21], as well as to each other. Lake Mina (45°53.40’N, 95°28.68’W) is located in an ecotone at the intersection of the northern coniferous-southern deciduous forest ecotone and the prairie-forest ecotone, and is therefore a sensitive site well-suited for paleoclimate reconstructions (S1 Fig.). Lake Mina is located 7 km to the east of Alexandria, Minnesota, which was reached by the railways in AD 1878. There were no signs of Native American agriculture in the pollen record (i.e., maize pollen) prior to Euro-American settlement. The ecotone consists of vegetation patches that contain all taxa that are present as pollen in the sediment core. Therefore response-lags caused by seed dispersal limitations are presumably non-existent, and vegetation response is relatively rapid [22]. The varve-dated core from Lake Mina spans AD 1116–2002 and is anchored by exotic pollen introductions and a basal radiocarbon date on micro-charcoal (see [22, 27] for varve chronology details). It was sampled at four-year continuous intervals for AD 1116–1952 and at two-year continuous intervals for the remainder. The data is available at the NOAA/World Data Center for Paleoclimatology Archive http://ncdc.noaa.gov/paleo/study/16761 and in the Neotoma Database http://www.neotomadb.org/.

Lake Mina’s February and May monthly mean temperature reconstructions from each calibration set were compared to each other and to the contemporaneous earliest instrumental climate records beginning in AD 1820 (Fig. 1). This 193-year continuous record for Alexandria is derived from Fort Snelling at Minneapolis-St. Paul, 195 km southeast of Lake Mina, and not from Alexandria. However, over this distance air temperatures are highly correlated (*r^2^_adj_* = 0.92 and *r^2^_adj_* = 0.88 for February and May mean temperatures between Fort Snelling and Alexandria), allowing linear regression-based estimations of Alexandria’s monthly mean temperature records for AD 1820–1885. These estimations, together with the continuous climate record for Alexandria that begins in AD 1886, forms the “extended Alexandria” record.

Analog analysis and a closer examination of calibration set bias were then performed in order to further explore which calibration set is most appropriate for paleo-climate inferences. Analog analysis with the squared chord distance metric was used to compare the Lake Mina pre-settlement pollen spectra to the 1870 and modern pollen sets to determine which approach best matched the sedimentary pollen record [28]. To further examine the bias induced by human disturbance, we then applied the modern pollen-climate calibration set to the pre-settlement pollen data set to reconstruct February and May monthly mean temperatures, which were then compared to the earliest dense instrumental climate records from AD 1895–1924 in order to determine if any systematic bias was present. For comparison, a parallel leave-one-out cross-validation analysis used the 1870 pollen-climate calibration set to make reconstructions for each pollen sample in the pre-settlement 1870 dataset [29]. Because *Ambrosia* and Poaceae are known indicators of human disturbance, they are often excluded from analysis in actual practice (e.g., [30]). Therefore, the entire analysis was repeated with these two taxa omitted.

## Results

### Changes in Minnesota climate and vegetation over the past 200 years

**Figure 2:**
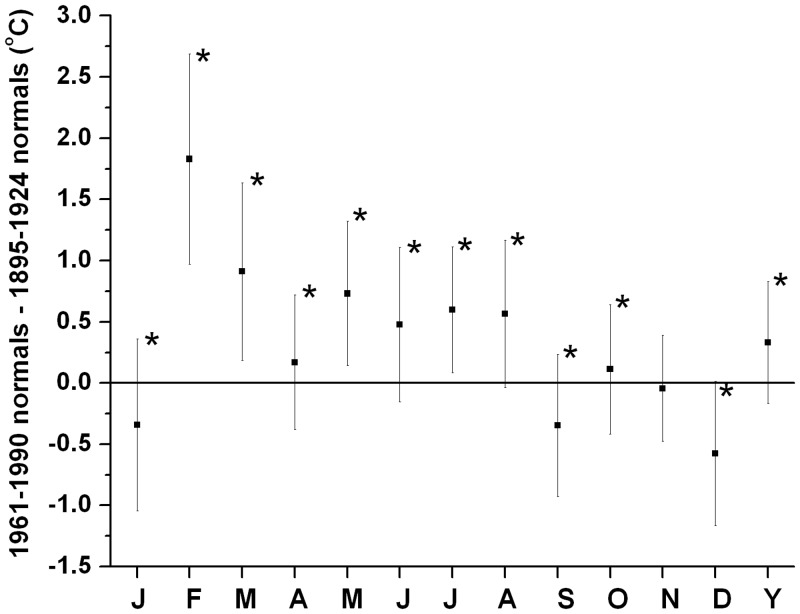
Comparison of the monthly climate normals used in the two calibration sets. The differences and standard deviations between the monthly mean temperatures of AD 1961–1990 and those of AD 1895–1924 are shown. Averages calculated over the 133 sites. An (*) denotes a significant change at the 0.05 level as assessed by a paired *t*-test with 132 degrees of freedom.

The climate of Minnesota has significantly warmed over the past two centuries. The Fort Snelling mean annual temperature record shows a temperature increase of 2.0°C from AD 1820 to AD 2012, based upon ordinary-least-squares regression analysis (Fig. 1). At Fort Snelling, monthly mean temperatures have significantly increased from AD 1840–1869 to AD 1961–1990 for all months except for January (Table 1). There were no significant monthly mean temperature differences between AD 1840–1869 and AD 1895–1924 for January-March and May-August (Table 1). The *t*-statistics are higher for the annual temperature normals than for the monthly normals because the annual temperatures have less variance than the monthly data. When the climate normals used in the two calibration sets are compared, the annual mean temperature and the monthly mean temperatures for February through August and October for AD 1961–1990 are significantly greater those of AD 1895–1924 (Fig. 2).

**Table 1:** Fort Snelling, Minnesota, USA, monthly climate normals comparisons.

**Time period**	**1895–1924 mean—1840–1869 mean**	***t-stat***	***p-level***	**1961–1990 mean—1840–1869 mean**	***t-stat***	***p-level***
**January**	1.21	1.28	0.21	1.33	1.38	0.17
**February**	-0.19	-0.23	0.82	**2.07**	**2.46**	**0.02**
**March**	1.33	1.34	0.19	**2.47**	**2.50**	**0.02**
**April**	**1.46**	**2.32**	**0.02**	**1.99**	**3.27**	**0.002**
**May**	0.62	1.18	0.24	**1.57**	**2.88**	**0.006**
**June**	0.46	1.01	0.32	**1.22**	**2.74**	**0.008**
**July**	0.34	0.78	0.44	**1.16**	**2.84**	**0.006**
**August**	0.71	1.89	0.06	**1.48**	**4.18**	**0.0001**
**September**	**1.52**	**3.00**	**0.004**	**1.64**	**3.52**	**0.0008**
**October**	**1.87**	**3.22**	**0.002**	**2.45**	**4.69**	**1.7 × 10^-05^**
**November**	**1.70**	**2.58**	**0.01**	**2.15**	**4.02**	**0.0002**
**December**	**2.18**	**2.74**	**0.008**	**1.90**	**2.50**	**0.02**
**Annual**	**1.14**	**4.34**	**5.7 × 10^-05^**	**1.81**	**6.81**	**6.2 × 10^-09^**

Shown are the differences between the AD 1895–1924 temperature means minus the AD 1840–1869 temperature means, and the AD 1961–1990 temperature means minus the AD 1840–1869 temperature means for each month and annually. Temperatures in °C; two-sided *t*-tests assuming equal variances, with 58 degrees of freedom; bold denotes significance at the 0.05 level.

**Figure 3:**
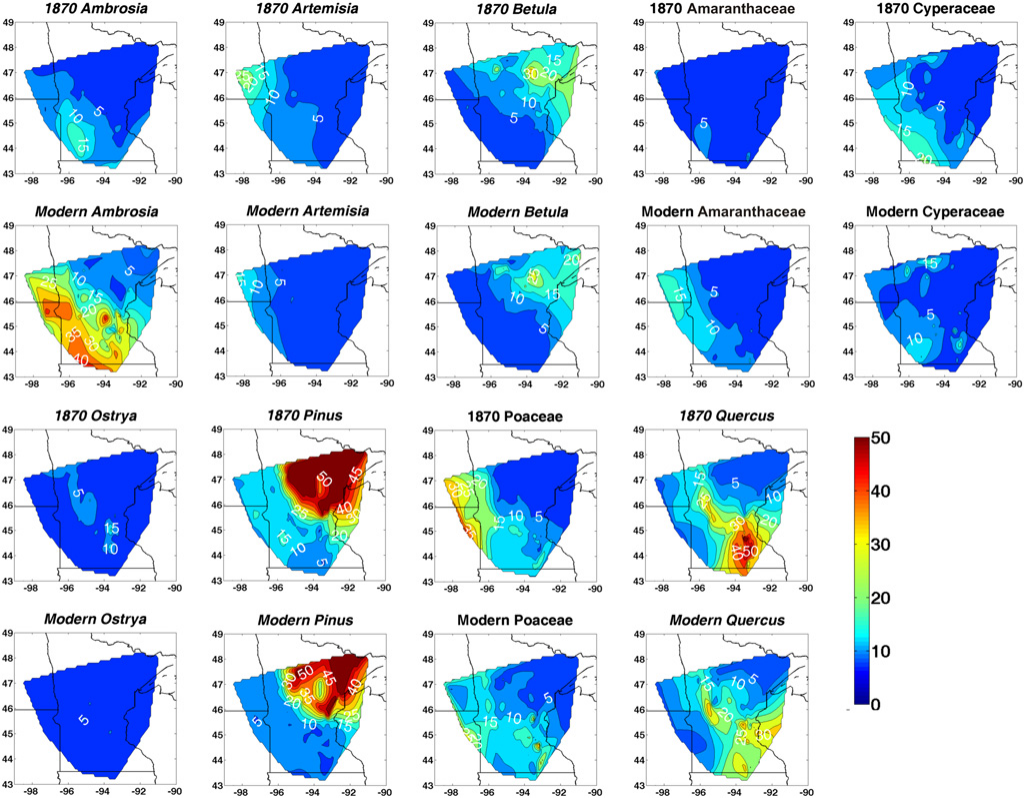
Minnesota isopoll maps from the pre-settlement AD 1870 and modern core-top samples. The nine taxa showing the greatest changes in relative abundance (%) after Euro-American settlement according to St. Jacques et al. [5] are presented. Interpolation based on natural neighbor interpolation (Matlab 2012).

The vegetation of Minnesota has been greatly altered over the past century and a half by direct human impacts from Euro-American settlement and climate change. *Ambrosia* and Amaranthaceae (including the subfamily Chenopodioideae) pollen have increased in relative abundance due to increased land clearance for agriculture; whereas *Artemisia, Betula*, Cyperaceae, *Ostrya, Pinus*, Poaceae and *Quercus*, characteristic of the native prairies and forests, have declined in relative abundance (Fig. 3).

### Lake Mina reconstructions

**Figure 4:**
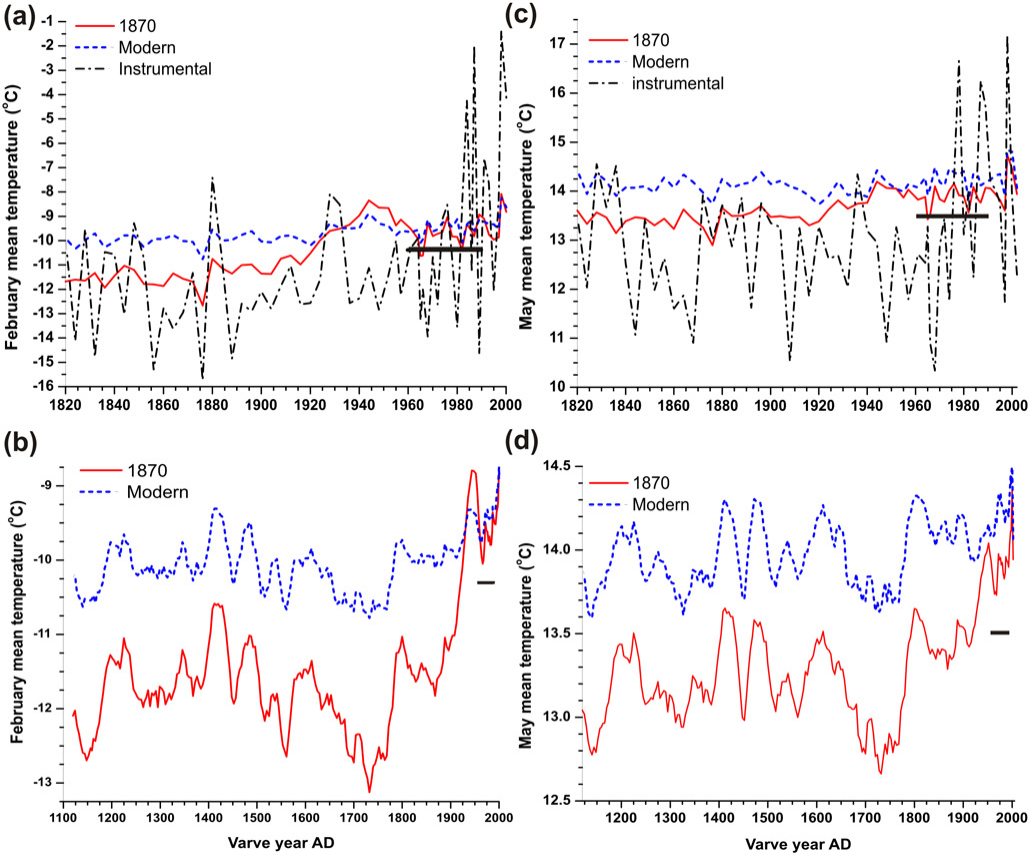
Lake Mina, Minnesota, pollen-inferred climate reconstructions using the pre-settlement 1870 and the modern calibration sets. (a) unsmoothed February mean temperatures for AD 1820–2002; (b) February mean temperatures for AD 1116–2002, smoothed by a 5-point moving average; (c) unsmoothed May mean temperatures for AD 1820–2002; and (d) May mean temperatures for AD 1116–2002, smoothed by a 5-point moving average. Also shown in (a) and (c) are the extended instrumental February and May mean temperatures for nearby Alexandria, Minnesota, AD 1820–2002, cumulatively averaged as the pollen samples were according to the varve counts for comparison. The black bars denote the AD 1961–1990 monthly climate normals.

Lake Mina pollen-inferred temperature reconstructions based upon the 1870 and the modern calibration sets were compared to the extended Alexandria instrumental records (AD 1820–2002) (Fig. 4). In order to make them comparable, the extended Alexandria February and May instrumental mean monthly records were averaged for the same years as the individual pollen samples, based on the varve counts. Student’s *t*-tests on the means and *F*-tests on the variability were performed between the reconstructions and the instrumental records for AD 1820–1876, as this period prior to the arrival of the railroads should contain minimal human disturbance. The climate reconstructions based upon the 1870 calibration set match the extended Alexandria instrumental climate record much more closely than the reconstructions based upon the modern calibration set (Fig. 4a and 4c).

For February, the 1870-based reconstruction has a mean of -11.6°C for AD 1820–1876, which is not significantly different from the mean (-12.3°C) of the extended Alexandria February instrumental record (Table 2). The 1870 reconstruction is not as variable as the averaged actual instrumental data (Table 2). This occurs because of slower-acting processes of vegetation composition and pollen production, the fact that the pollen calibration set was compiled from 30-year climate normals, and because, in any annual varve, there is pollen washing in from previous years, so some smoothing of the pollen record occurs (see discussion for fuller treatment). However, the modern-based February reconstruction mean is -10.0°C for AD 1820–1876, which is significantly warmer than the instrumental mean and the 1870 reconstruction mean (Table 2). The modern reconstruction is also less variable relative to the actual instrumental data (Table 2). The difference between the variabilities of the modern and 1870 reconstructions is not significant.

**Table 2:** Comparisons of the reconstructions to each other and the early instrumental data.

**Means**	***µ_1_***	***µ_2_***	***t-statistic***	***d.f.***	***p-value***
**February reconstruction**					
**1870 vs instrumental**	-11.6	-12.3	1.11	15	0.28
**Modern vs instrumental**	-10.0	-12.3	3.96	14	**0.001**
**1870 vs modern**	-11.6	-10.0	-13.09	26	**6.0x10^-13^**
**May reconstruction**					
**1870 vs instrumental**	13.4	12.8	2.03	15	0.06
**Modern vs instrumental**	14.1	12.8	-4.44	15	**0.0004**
**1870 vs modern**	13.4	14.1	-10.6	28	**2.7x10^-11^**

**Variability**	***σ_1_***	***σ_2_***	***F-statistic***	***d.f.***	***p-value***
**February reconstruction**					
**1870 vs instrumental**	0.38	2.17	0.031	14,14	**3.3x10^-8^**
**Modern vs instrumental**	0.28	2.17	0.017	14,14	**5.1x10^-10^**
**1870 vs modern**	0.38	0.28	0.538	14,14	0.13
**May reconstruction**					
**1870 vs instrumental**	0.19	1.17	38.2	14,14	**1.1x10^-8^**
**Modern vs instrumental**	0.19	1.17	37.2	14,14	**1.3x10^-8^**
**1870 vs modern**	0.19	0.19	2.16	14,14	0.08

Student’s *t*-tests and *F*-tests of the AD 1820–1876 means (*µ_1,_ µ_2_*) and standard deviations (*σ_1_, σ_2_*) of the February and May 1870-based and modern-based reconstructions and the annual instrumental temperatures, the latter averaged as the pollen samples were according to the varve counts. Bold denotes significance at the 0.05 level.

For May, the 1870-based reconstruction has a mean of 13.4°C for AD 1820–1876, which is not significantly different from the mean (12.8°C) of the extended Alexandria instrumental record (Table 2). Again, the May 1870-based reconstruction is not as variable as the actual averaged instrumental data (Table 2). The modern-based May reconstruction is warmer with a mean of 14.1°C for AD 1820–1876, which is significantly different from the instrumental mean and the 1870 reconstruction mean (Table 2). The modern reconstruction is also less variable relative to the actual instrumental data (Table 2). There is no significant difference between the variability of the modern-based reconstruction and that of the 1870 reconstruction (Table 2).

The discrepancies arising from the two different calibration sets persist for the last millennium (Fig. 4b and 4d). The February 1870-based reconstruction has a mean of -11.8°C for AD 1116–1876, whereas the modern-based reconstruction has a mean of -10.1°C—a difference of 1.7°C. The May 1870-based reconstruction has a mean of 13.2°C for AD 1116–1876, whereas the modern-based reconstruction has a mean of 14.0°C—a difference of 0.8°C. Furthermore, greater variance for both the February and May temperatures during this period is shown by the 1870-based reconstructions than by the modern-based reconstructions (*F* = 2.21; *d.f.* = 190,190; *p-value* = 3.8 × 10–^8^; and *F* = 1.35; *d.f.* = 190,190; *p-value* = 0.02, respectively). As an estimate of the regional warming since the LIA due to anthropogenic climate change, the differences between the instrumental 1961–1990 February and May normals and the AD 1450–1850 mean reconstructions can be used. The February 1870-based reconstruction shows a rise of 1.6°C and the May 1870-based reconstruction shows a rise of 0.3°C. The February modern-based reconstruction shows no temperature change and the May modern-based reconstruction shows a decline of -0.5°C. Hence, choice of calibration set can have a large impact on centennial-scale temperature reconstructions.

### Analog analysis

**Figure 5:**
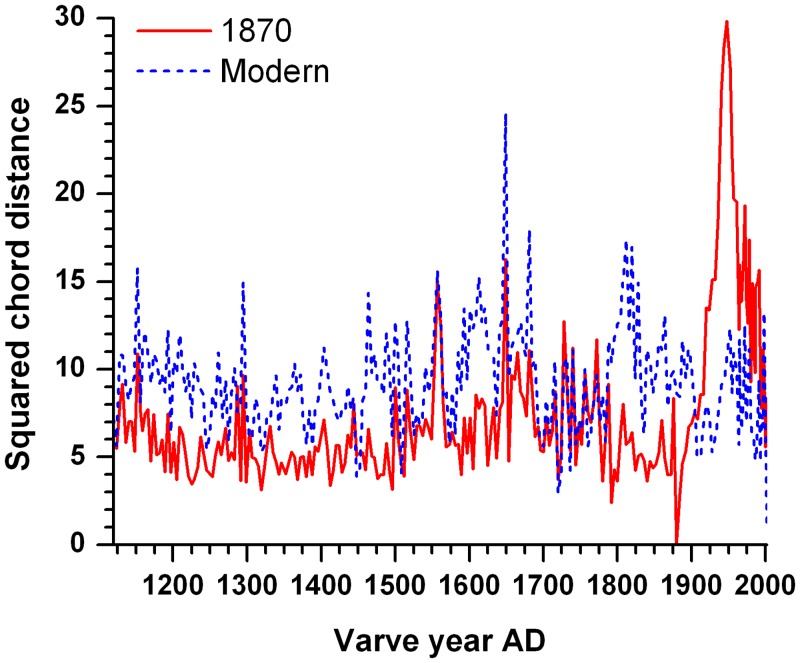
Analog analysis. Diagram showing minimum squared chord distances between the Lake Mina pollen samples (AD 1116–2002) and their nearest neighbors in the pre-settlement 1870 pollen calibration set and the modern pollen calibration set.

The minimum squared chord distances between the Lake Mina pollen samples and their nearest neighbors in the 1870 and modern sets show that the 1870 calibration set provides better analogs to the Lake Mina pollen assemblages from AD 1116–1876 than the modern set (Fig. 5). For all samples prior to 1900, the 1870 set provides analogs with a mean chord distance of 6.1, whereas the modern set provides analogs with a mean distance of 9.4, an increase of 55%. The 1870 set has poor analogs after AD 1900 when agricultural usage intensified, until AD 1993 when it has close analogs again due to increasing reforestation.

### Hindcasting results

**Figure 6:**
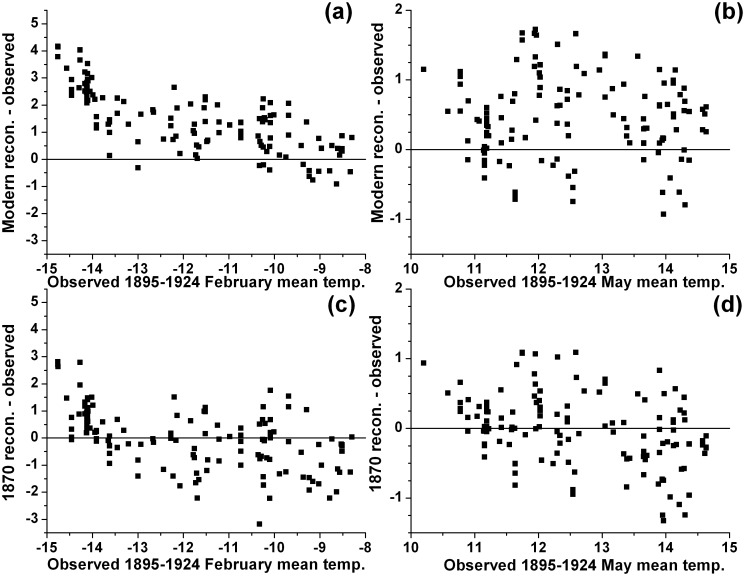
Residuals of the reconstructed 1870 pollen sets from hindcasting and cross-validation. The reconstructions from hindcasting (i.e., the application of the modern calibration set to the pre-settlement 1870 pollen samples for each of the 133 sites) minus their paired observed AD 1895–1924 temperatures (°C) for (a) February and (b) May are shown. Also shown are the reconstructions from leave-one-out cross-validation (i.e., the application of the pre-settlement 1870 calibration set to the 1870 pollen samples for each of the 133 sites) minus their paired observed AD 1895–1924 temperatures (°C) for (c) February and (d) May.

We used the modern pollen-climate training set to reconstruct February and May mean monthly temperatures for each of the 133 samples in the 1870 pollen set. Fig. 6a and 6b show the residuals between the reconstructed climate values and the observed values from the AD 1895–1924 climate dataset, which are assumed to be reasonable estimators of the climate of AD 1840–1870, the estimated dates of the pre-settlement pollen (Table 1). The residual plots show consistently high positive bias for both February and May, i.e., temperatures for both months are overestimated. The mean positive bias is 1.5°C for February and 0.5°C for May. For comparison, the residual plots from the leave-one-out cross-validation analysis (Fig. 6c and 6d) show a bias of -0.006°C for February and 0.0005°C for May. Although the residual standard deviations are very similar for both the hindcasting and cross-validation analyses (for February 1.1°C versus 1.1°C, and for May 0.6°C versus 0.5°C), the hindcasting residuals have a more non-random structure. Hence, using a modern pollen-climate dataset, rather than a pre-settlement calibration set, introduces a mean positive bias of at least 0.5–1.5°C.

Another way to determine if the 1870 calibration set performs better than the modern set is to examine whether the error when the modern transfer function is applied to the 1870 pollen set (i.e., when hindcasting) is larger than the cross-validation error of the 1870 data set (i.e., when applying the 1870 transfer function to the 1870 pollen set). For February mean temperature, hindcasting with the modern transfer function results in a RMSE of 1.84°C, versus a root mean squared error (RMSE_app_) of 1.02°C and a bootstrapped root mean squared error of prediction (RMSEP_boot_) of 1.12°C from cross-validation, a bootstrapped improvement of 64% [31]. For May mean temperature, hindcasting results in a RMSE of 0.76°C, versus a RMSE_app_ of 0.51°C and a RMSEP_boot_ of 0.54°C from cross-validation, a bootstrapped improvement of 41%. Therefore, the 1870 calibration set again outperforms the modern calibration set.

For all of the above analyses, the modern calibration set with both *Ambrosia* and Poaceae deleted, and the modern calibration set with *Ambrosia* alone deleted, gave virtually identical results to the original modern set and are not shown further. Therefore, the practice of deleting these two taxa in order to remove the effects of human disturbance does not alleviate this issue.

## Discussion

In the upper Midwest of the United States, significant low-frequency signal attenuation and warm bias of 0.8–1.7°C exists when using a conventional modern pollen-climate calibration set, rather than a pre-settlement pollen-climate calibration set, to infer paleotemperatures. For paleoclimate reconstructions in Minnesota, this can result in an overestimation of Little Ice Age temperature and consequently an underestimation of the extent of regional warming. Therefore, we suggest that any past pollen spectra from before Euro-American settlement in this region should not be interpreted using modern pollen-climate calibration sets, as these sets contain a substantial anthropogenic bias. Rather, pre-1900 reconstructions should ideally be interpreted using a pre-Euro-American settlement pollen set, paired to the earliest instrumental climate records. The size of this signal attenuation and bias is of the same order of magnitude as the amplitude between maximum and minimum temperatures during the past two millennia on centennial timescales in the individual records in papers such as [7] and [8]. These individual records show an average difference of 1.5–2°C, with many proxy records showing less [14]. Hence, there is reason for concern for those doing pollen-based climate reconstructions of the past two millennia, at least from regions of heavy human impacts such as North America and Europe. Efforts should be made to evaluate the extent and severity of problems associated with the use of modern pollen-climate calibration sets and the modern analog technique (MAT) to reconstruct North American temperatures over the past millennium (e.g., [10, 32, 33]).

An assessment of the accuracy and bias of pollen-inferred climate records is important because such records are widely used to reconstruct past climates and to evaluate the simulations of earth system models. High-resolution pollen counts can be used to produce valuable terrestrial paleoclimate records, especially from regions where results from other proxies are lacking, such as the Great Plains of North America, which is unsuitable for tree-ring temperature reconstructions [9, 22]. High-resolution pollen records poorly capture high-frequency (annual-scale) climate variance as seen in this study, in part because of the use of 30-year climate normals in constructing the transfer functions (Fig. 4) [34]. Only 18% (16%) of the total instrumental variance for AD 1820–1876 is captured in our February (May) reconstruction. However, high-resolution pollen data do record low-frequency (multi-decadal) variability [2, 34]. Because of this, high-resolution pollen records are being used to build stacked paleoclimate reconstructions that combine low-frequency pollen records with high-frequency tree-ring records [7, 10, 34]. A further use of high-resolution pollen data is to extend shorter tree-ring studies, particularly to cover the first millennium AD, where tree-rings become relatively sparse. Less variance in some pollen records than in same-resolution tree-ring records has been observed [9, 10]. As shown in this study, some of this variability loss could be a result of using a conventional modern pollen-climate calibration set which introduced low-frequency signal attenuation. To the best of our knowledge, this paper is one of the few studies (see also [5, 6]) that examines the non-stationarity of the pollen-climate relationship between the present with its highly disturbed landscapes and the less-disturbed past from which we wish to derive climate inferences. Unlike tree-ring studies, pollen-based transfer functions are calibrated over space rather than time, and there is little practice of then validating the pollen-based reconstruction against early long instrumental records (but see [34, 35]). This lack of validation needs to be critically re-examined.

Additionally, high-resolution pollen reconstructions are a method to validate tree-ring and other proxy reconstructions, as inter-comparisons among proxies are the only practical way to validate a paleoclimate reconstruction over multi-centennial timescales. Many of the high-resolution climate reconstructions of the past two millennia that show steep anthropogenic warming have been based upon tree-rings, as the period of instrumental climate records is of such short duration [9, 11, 14]. However, tree-ring reconstructions typically suffer from the loss of low-frequency variability from short segment lengths [36]. High-resolution pollen analysis, relatively free of bias and low-frequency signal attenuation, is one possible tool to validate tree-ring reconstructions and specifically to assess the effectiveness of methods of preserving low-frequency variance, such as Regional Curve Standardization and Age Band Decomposition [37–39]. From pseudo-proxy studies, among others, there is concern that low-frequency variance is attenuated in millennial paleoclimate temperature reconstructions [15–17]. Some of this signal attenuation could be due to the heavy reliance of the hemispheric temperature reconstructions on annually resolved tree-ring data. Hence, any means of assessing how well methods preserve low-frequency variability in tree-rings is worthwhile.

Our analysis relies upon the Lake Mina pollen record to be relatively unlagged in its response to changing climate and to reasonably capture the climate variability in the 20–1000 year band, as we are comparing 57-year mean pollen-inferred temperatures to contemporaneous mean instrumental temperatures. Much of the pollen literature (e.g., [40, 41]) assumes that it is primarily by seed migration and growth to maturity from seed that terrestrial vegetation can record the effects of climate via the deposition of pollen in lacustrine sediments. This necessarily would entail a lagged response in the Lake Mina pollen record of at least ~50 years for trees to grow to sexual maturity, assuming that all species are present as seed in the broad, diverse ecotone. However, vegetation can show a vegetative response to weather that can also be identified in a pollen record. There is a growing literature showing that pollen production responds to current year’s or previous year’s weather, especially when species are at their range limits as are many dominant species at Lake Mina (e.g., [35, 42–47]). Fast vegetative responses can occur via the number of male cones initiated in conifers, the number of flowers initiated in angiosperms, the number of branches, and the number of annual plants flowering, etc. Changes in the pollen accumulation rates will be reflected in changes in the pollen percentages preserved in sediments since it is highly unlikely that each species will vary its pollen production in the same way according to weather.

The swift response of the Lake Mina pollen record to changing weather/climatic conditions can be explicitly examined because there are three other independent proxy climate records from the lake: varve thickness, and diatom and chrysophyte microfossils from split samples [22, 26]. The varve thickness series records the 1300s Mississippian megadrought [48] (as thick light-colored sandy sediments) as occurring from AD 1320–1385. In the Lake Mina pollen record, the second principal component (PC2) records the prairie pollen taxa and is hence a drought indicator [22]. PC2 shows the Mississippian megadrought to have a sharp beginning in the interval AD 1316–1319 and to have ended at AD 1384–1387. Here pollen may be able to record a quicker response than varve thickness because the lake probably needed time to expose the sub-littoral sandy sediments which form these distinct varves. The diatom valves and chrysophyte scales show a major reorganization of the lake at the onset of the LIA, which presumably arose because of weather pattern changes [26]. These algae have a fast same-year response to changing conditions. The diatom:chrysophyte scale ratio (where low values show when spring and autumn circulation periods were short and seasonal transitions to stratified conditions were rapid and severe, high values the converse) changes from consistently high values in the MCA to consistently low values in LIA at AD 1469–1472. The diatom first principal component (PC1) (a metric of the intensity of spring mixing) changes from frequent high values in the MCA to relatively low values in LIA at AD 1477–1480. Unfortunately, neither the diatom:chrysophyte scale ratio nor diatom PC1 are recording temperature *per se,* which makes comparisons to the pollen record less direct, but presumably these large weather pattern shifts were antecedent to or accompanied the cooler temperatures of the LIA. The pollen PC1 (a metric of temperature) shows the initiation of the LIA at AD 1505–1508. Hence, there is a 0–35 year lag in the pollen response at Lake Mina, depending on the type of climate change. This lag is short enough to make our analysis valid. Furthermore, our analysis does not need the full reconstruction of the annual high-frequency band in the climate record. A reasonable capturing of the variability in the 20–1000 year band with some of the annual variability is sufficient, and we argue that the Lake Mina 1870-based temperature reconstructions contain this.

Independent evidence from borehole annual temperature reconstructions supports the reconstructions based upon the pre-settlement calibration set, but not those based upon the conventional set. Borehole studies from the adjacent prairie provinces of Canada have shown a regional warming of about 1.5–2.5°C over the past 300 years [49–51]. Direct comparisons to the differences between the Lake Mina late 20^th^ century pollen-based temperature reconstructions and 18^th^ century reconstructions are not possible because the 20^th^ century reconstructions are unreliable due to landscape alterations. However, the differences between the instrumental Alexandria monthly climate normals of AD 1961–1990 and the average monthly temperature reconstruction for the 18^th^ century can be calculated for both February and May. The mean difference from both months can be used as an estimate of the annual temperature change during the past 300 years. For the pre-settlement reconstruction, the difference was 1.3°C; whereas the difference was -0.1°C for the modern reconstruction. The pre-settlement reconstruction matches the borehole temperature changes well; the modern reconstruction does not match the borehole reconstructions.

Although not perfect, the 1870 calibration set captures the pre-settlement relationship between the pollen rain and climate better than the modern set. The pollen data reflect the pre-Euro-American vegetation controlled by climate and other natural processes relatively undisturbed by humans, unlike the most recent (core-top) pollen spectra, which is an integration of both climate and land use. Contemporaneous lake ice-out and ice-in records [52] show that the Fort Snelling temperature record is broadly representative of Minnesota. There were no significant monthly mean temperature differences between AD 1840–1869 and AD 1895–1924 for January-March and May-August (Table 1). Because of this, and given the homogeneity of temperature fields, the climate normals of AD 1895–1924 for the 133 sample sites are reasonable estimators for those of AD 1840–1869, although they are slightly warmer (Table 1).

On the other hand, a modern pollen-climate calibration set has some problems. The most important is that the pollen data comes from vegetation typically heavily modified by human impacts. Furthermore, not all locales have received the same amount of direct human impact. Due to the rapidity of anthropogenic global warming, vegetation is likely lagging behind climate and not all pollen taxa are responding to the rapid global warming at the same rate. A modern pollen calibration set is typically drawn from a compiled mixture of present-day surface samples and core-top samples, the latter of which can date from anytime in the past hundred years or so (because not all researchers conserved the sediment-water interface), and not from the exact three decades that the climate normals are computed from. Our study shows that, if we want to use high-resolution pollen in millennial climate reconstructions, it is important to construct our transfer functions more carefully and address these known, but overlooked issues.

Theoretically, a conventional modern core-top pollen-climate calibration set could produce systematically biased reconstructions in either a colder or warmer direction because both Minnesota’s vegetation and climate have been anthropogenically modified. First, the bias could be towards a colder direction because, due to landscape disturbance, the modern pollen set based upon core-tops could typically have more thermophilic and ruderal taxa such as *Ambrosia* and Amaranthaceae pollen, at the expense of northern taxa such as *Pinus* and *Betula* (Fig. 3) [5]. Hence, the modern vegetation artificially appears to be from a warmer climate than it should be relative to the AD 1961–1990 climate normals. This would have the effect of mapping the pre-settlement vegetation to a cooler climate than it experienced. Hence, one would expect negative mean residuals between the reconstructed 1870 temperatures using the modern calibration set applied to the 1870 pollen minus their paired observed AD 1895–1924 temperatures (Fig. 6), and the modern reconstructions should be biased towards being too cold (Fig. 4a and 4c). Yet this is not the case because the bias could also be towards warmer reconstructions. Because of the twentieth century’s rapid rate of warming [53], climate and vegetation are out of equilibrium, with a vegetation typical of a cooler climate lagging a warming climate. This would have the effect of mapping the vegetation of AD 1870 to a warmer climate than it actually experienced. This effect is re-enforced by landscape disturbance, since the modern pollen set based upon core-tops has less of the warmer *Quercus, Ostrya, Artemisia,* Poaceae and Cyperaceae pollen than it should have naturally (Fig. 3) [5]. This latter landscape modification re-enforces the tendency of mapping the vegetation of AD 1870 onto a warmer climate. Hence, the mean residuals between the reconstructed Minnesota 1870 temperatures minus their observed AD 1895–1924 temperatures would be positive, as they are (Fig. 6). Therefore, it appears that the tendency towards bias in a warmer direction dominates in the reconstructions based on the conventional modern calibration set (Figs. 4a, 4c and 6) over the competing tendency in a colder direction. This is indeed observed with the modern reconstructions, i.e., they are too warm relative to the instrumental data of the 1800s.

## Conclusions

We examine the effect of direct and indirect modern human landscape disturbances on a conventional pollen-climate calibration set used to infer paleoclimate reconstructions from a pre-disturbance pollen record. We show that warm bias and low-frequency signal attenuation are significant in a conventional pollen record from the north central United States. This causes the amount and rate of regional manifestations of global warming to be significantly underestimated when using a conventional pollen analysis. It would be illuminating for palynologists to perform studies similar to this one from other suitable regions to determine how widespread this bias and low-frequency signal attenuation problem is in pollen reconstructions. The chain of early US military forts from the Canadian border to the Gulf coast, many of which were founded in the 1820s, is one possible source of early climate data (e.g., [54]).

## Supporting Information

S1 Fig Location of Lake Mina, Minnesota. Map of Minnesota and environs showing locations of Lake Mina (Mi), Fort Snelling (FS) and the main vegetation patterns.
(TIF)
