# Supplementary figures and images for: The Bias and Signal Attenuation Present in Conventional Pollen-Based Climate Reconstructions as Assessed by Early Climate Data from Minnesota, USA
Source: PLoS One. 2015 Jan 20;10(1):e0113806. doi: 10.1371/journal.pone.0113806 (PMC4300216; doi:10.1371/journal.pone.0113806)

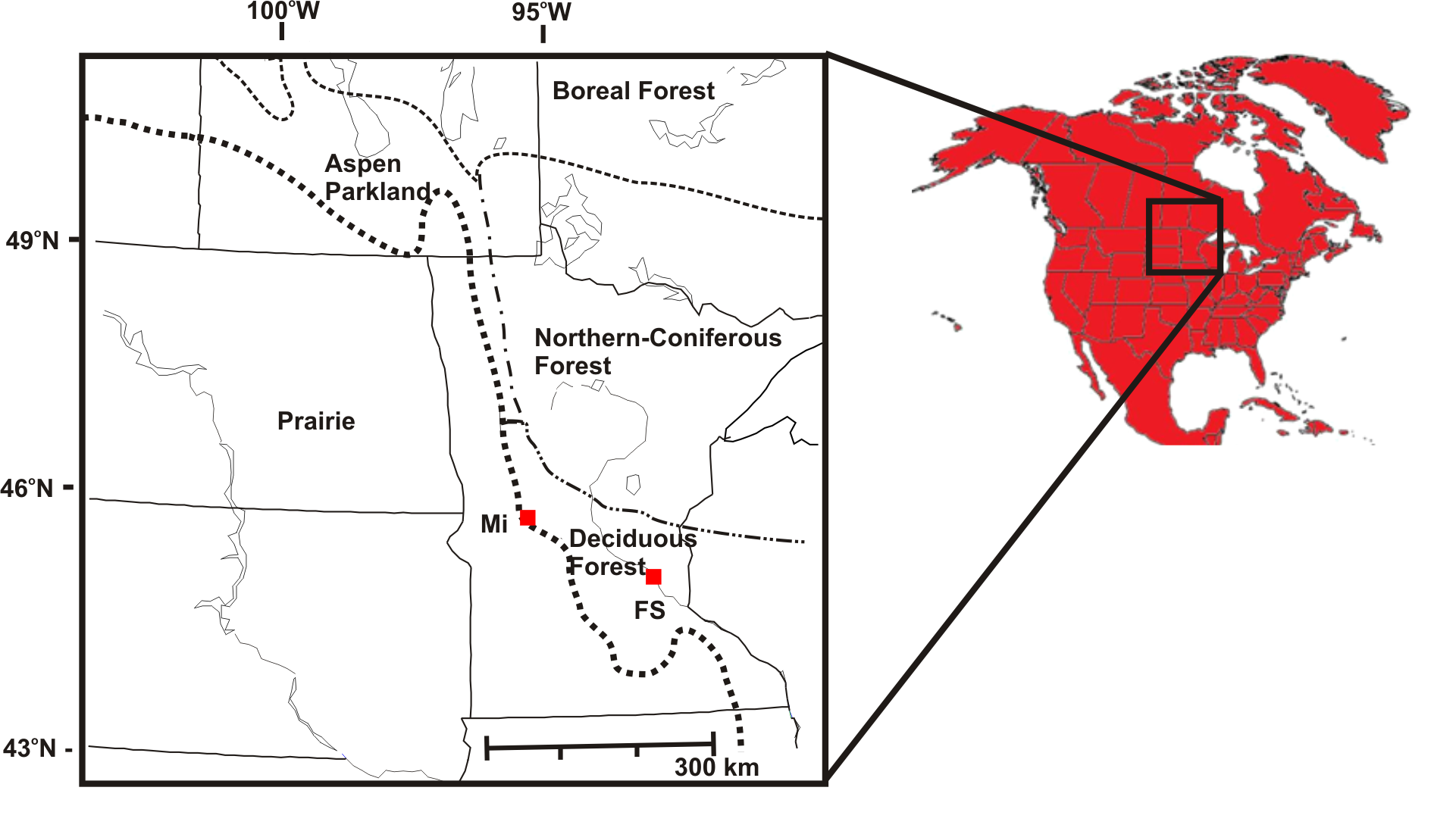

Supplement: S1 Fig — Map of Minnesota and environs showing locations of Lake Mina (Mi), Fort Snelling (FS) and the main vegetation patterns. (TIF) [file pone.0113806.s001.tif]
